# Oncologic outcomes following neoadjuvant immunochemotherapy in locally advanced oral squamous cell carcinoma

**DOI:** 10.3389/fimmu.2025.1571285

**Published:** 2025-05-08

**Authors:** Gang Li, Jiheng Wang, Qigen Fang, Liyuan Dai, Wei Du

**Affiliations:** Department of Head Neck and Thyroid, The Affiliated Cancer Hospital of Zhengzhou University & Henan Cancer Hospital, Zhengzhou, China

**Keywords:** neoadjuvant immunochemotherapy, oral squamous cell carcinoma, chemoradiation, radiotherapy, safety

## Abstract

**Background:**

To assess the oncologic outcomes in patients with oral squamous cell carcinoma (SCC) who underwent treatment with radiotherapy (RT) or chemoradiation therapy (CRT) following neoadjuvant immunochemotherapy and surgery.

**Methods:**

Data from patients who underwent neoadjuvant immunochemotherapy, surgery, and adjuvant therapy were collected prospectively and analyzed retrospectively. The primary outcomes assessed were 3-year overall survival and locoregional control. Secondary endpoints included the objective response rate (ORR), rates of pathologic complete response (pCR) and major pathologic response (MPR), as well as safety.

**Results:**

A total of 137 patients were included in the analysis. Neoadjuvant therapy yielded an ORR of 81.7%, with pCR and MPR achieved in 47 and 73 patients, respectively. Grade III and IV adverse events were rare, comprising only 1.6% of all events. The addition of adjuvant chemotherapy to RT did not show a significant reduction in the risk of locoregional recurrence. However, with regards to overall survival, the hazard ratios were 0.85 (95% CI: 0.73-0.96) for the MPR group and 0.66 (95% CI: 0.37-0.89) for the pCR group, both significantly higher than that in patients with incomplete pathologic response. The addition of adjuvant chemotherapy to RT was associated with a 5% reduction in the risk of mortality (95% CI: 1%-14%), the protective effect of CRT was the most obvious in patients with MPR.

**Conclusion:**

Neoadjuvant immunochemotherapy demonstrated high safety and efficacy in oral SCC. CRT was superior to RT in terms of overall survival especially in patients with MPR when administered following neoadjuvant immunochemotherapy and surgery.

## Background

Oral squamous cell carcinoma (SCC) represents the predominant histological subtype among head and neck malignancies, often presenting at an advanced local stage upon initial detection (1). The standard therapeutic approach typically involves a combination of surgical intervention and adjuvant radiotherapy (RT). However, despite advancements in reconstructive techniques utilizing regional and free flap procedures, the profound impact of vital organ resection on the quality of life remains a significant concern in clinical practice (2, 3).

While traditional neoadjuvant chemotherapy regimens centered around platinum agents have not shown a significant survival advantage in oral SCC (4), they have been linked to a substantial increase in the possibility of preserving the mandible by nearly 50% (5). With a deepening understanding of immune checkpoint pathways, immunotherapy has emerged as a superior alternative to traditional chemoradiotherapy, leading to prolonged overall survival in recurrent or metastatic head and neck SCC (6, 7). Nivolumab and pembrolizumab has been approved by FDA in SCC in head and neck (8). The integration of immunotherapy into neoadjuvant protocols has garnered considerable interest, with a series of clinical trials demonstrating that neoadjuvant immunotherapy, with or without chemotherapy, can achieve an impressive objective response rate (ORR) exceeding 95%. Moreover, pathologic complete response (pCR) rates of 30% or higher and major pathologic response (MPR) rates of approximately 70% have been observed (9, 10). These compelling outcomes prompt a reevaluation of the optimal management approach for oral SCC patients who achieve pCR or MPR following neoadjuvant immunotherapy.

Against this backdrop, the present study aims to assess the oncologic outcomes in oral SCC patients who have undergone treatment with radiotherapy (RT) or chemoradiation therapy (CRT) following neoadjuvant immunochemotherapy and surgery.

## Patients and methods

### Ethical approval

This study was approved by Henan Cancer Hospital Institutional Research Committee, and written informed consent for medical research was obtained from all patients before starting the treatment. All methods were performed in accordance with the relevant guidelines and regulations.

### Study design

In pursuit of this objective, prospectively collected data was subjected to retrospective analysis. Commencing in January 2019, a regimen combining immunotherapy and chemotherapy was implemented in neoadjuvant management of locally advanced oral SCC following thorough elucidation of potential complications. Between January 2019 and December 2022, a total of 154 patients diagnosed with primary locally advanced oral SCC underwent neoadjuvant immunochemotherapy, with subsequent surgical intervention performed on 137 patients who constituted the final cohort for analysis; 17 patients were excluded due to lack of surgical intervention. Comprehensive data encompassing demographic profiles, pathological characteristics, treatments administered, and follow-up details for these patients were meticulously documented.

### Variable definition

Assessment of all pathological sections was conducted by at least two specialized head and neck pathologists. Locally advanced disease staged as cT1-2N1-3 or cT3-4N0-3 was classified in alignment with the 8th edition of the AJCC system. Lymphovascular invasion (LVI) was deemed positive if cancer cells were detected within lymphatic vessels, while perineural invasion (PNI) was considered positive if cancer cells infiltrated a nerve. Extranodal extension (ENE) was indicative of cancer cells extending beyond the lymph node (LN) capsule. pCR denoted the absence of residual viable tumor cells in both the primary tumor and all resected lymph nodes, whereas MPR indicated ≤10% residual viable tumor cells in the resected tumor specimens. Incomplete pathological response (IPR) signified the presence of >10% viable tumor cells in resected tumor specimens. Immunohistochemical staining of PD-L1 expression was performed using the PD-L1 IHC 22C3 pharmDx assay with evaluation based on the combined positive score (CPS), determined by the number of PD-L1-staining cells divided by the total viable tumor count.

ORR was defined as the proportion of patients exhibiting a best response of complete or partial response as per RECIST 1.1 criteria before surgery (11). Clinical to pathological downstaging was characterized by a decline in T or N stage of pathologic staging relative to clinical staging (cTNM) according to the 8th edition of the AJCC cancer staging manual. Adverse events were graded in accordance with the National Cancer Institute Common Terminology Criteria for Adverse Events, version 4.0 (12).

### Outcome variables

Primary outcome variables encompassed 3-year overall survival (OS) and locoregional control (LRC), with OS time calculated from the date of surgery to the date of death or last follow-up, and LRC time calculated from the date of surgery to the date of initial locoregional recurrence or last follow-up. Co-secondary endpoints included ORR, rates of pCR and MPR, and safety markers.

### Treatment

Treatment protocols involved the administration of docetaxel at 75mg/m^2^ on days 1 and 8, cisplatin at 75mg/m^2^ on days 1 and 2, and pembrolizumab at 200mg on day 4 of each three-week cycle for two or three cycles. Surgery was scheduled within one to four weeks post completion of the six-week neoadjuvant regimen. Surgical plans and resection margins were predefined based on baseline evaluations preceding neoadjuvant therapy and remained unchanged irrespective of treatment response. Subsequent RT or CRT was initiated within six weeks post-surgery, targeting the tumor bed with a 1-2cm margin, and a prescribed dose of 60-66 Gy. Adjuvant chemotherapy was administered guided by clinical judgment and pathological characteristics, typically entailing cisplatin over 4-6 cycles at 75mg/m2.

### Statistical analysis

For primary outcome variables, the impact of RT versus CRT on OS and LRC was assessed using univariate and multivariable Cox models, with outcomes presented as hazard ratios (HR) and 95% confidence intervals (CI). Secondary endpoints were descriptively outlined. Statistical analyses were conducted using R 3.4.4, with a significance level set at p<0.05.

## Results

### Baseline data

A total of 137 patients (90 males and 47 females) were enrolled for analysis, with a mean age of 50 ± 18 years. Among the cohort, 80 patients were active smokers, and 61 individuals reported alcohol consumption. Primary tumor sites were categorically distributed as follows: 61 cases in the tongue, 31 in the floor of the mouth, 25 in the buccal region, and 20 in the gingiva. Clinical tumor staging revealed T2 tumors in 14 patients, T3 in 85, and T4 in 38 cases. Notably, 87 patients presented with clinically positive lymph nodes, with 29 cases classified as N1, 40 as N2, and 18 as N3. Cancer staging indicated stage III disease in 39 patients and stage IV in 98 individuals. Assessment of PD-L1 expression demonstrated a CPS of less than 1 in 32 patients and 20 or higher in 38 patients. All patients achieved negative surgical margins. Two and three cycles of neoadjuvant therapy were administered to 100 and 37 patients, respectively. of Among the cohort, 77 patients underwent treatment with RT, while the remaining received treatment via CRT. Both treatment groups demonstrated a harmonious distribution across these parameters (**Table 1**, all p>0.05).

**Table 1:** Baseline date of the 137 patients treated by neoadjuvant immunochemotherapy.

Variable	Total	RT (n=77)	CRT (n=60)	p*
Age				
≤50	81	47	34	
>50	56	30	26	0.606
Sex				
Male	90	50	40	
Female	47	27	20	0.832
Smoker				
Yes	80	40	40	
No	57	37	20	0.083
Drinker				
Yes	61	35	26	
No	76	42	34	0.804
Site				
Tongue	61	31	30	
Mouth floor	31	18	13	
Buccal	25	15	10	
Gingiva	20	13	7	0.674
cT				
T2	14	8	6	
T3	85	45	40	
T4	38	24	14	0.571
cN				
N0	50	30	20	
N1	29	17	12	
N2	40	22	18	
N3	18	8	10	0.706
Cancer stage				
III	39	24	15	
IV	98	53	45	0.427
CPS^%^				
<1	32	20	12	
1-19	67	37	30	
≥20	38	20	18	0.691

*Comparison between radiotherapy (RT) and chemoradiation (CRT) groups.

% CPS, combined positive score.

### Efficacy

**Figure 1:**
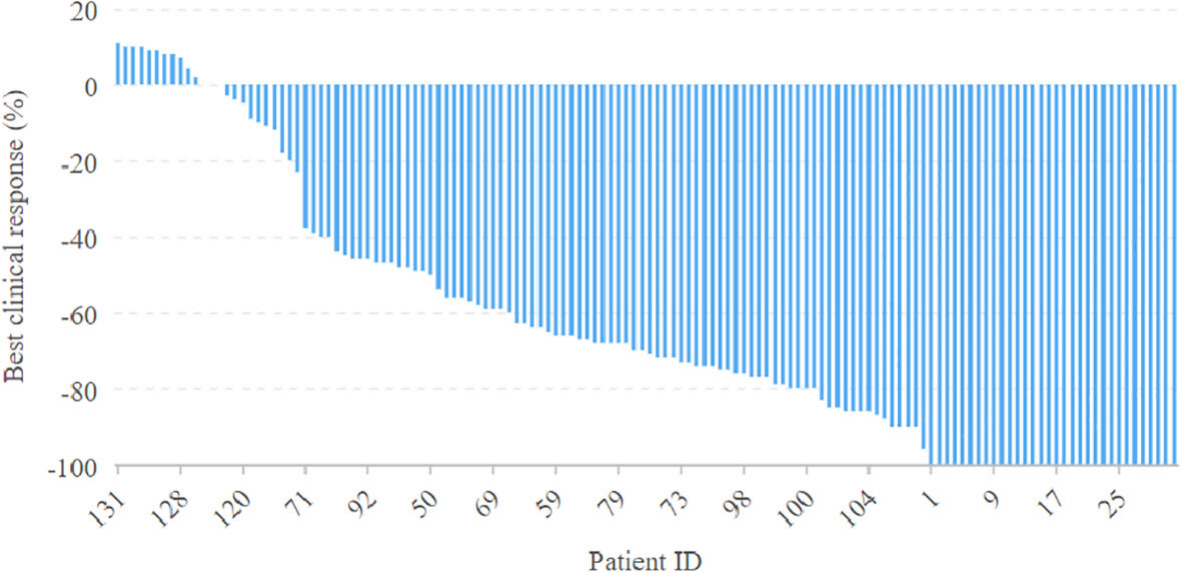
Clinical evaluation of neoadjuvant immunochemotherapy efficacy in the 137 patients with oral squamous cell carcinoma.

All patients completed the designated two cycles of neoadjuvant immunochemotherapy. Clinical evaluation revealed that 32 patients attained a complete response, 80 manifested a partial response, and 25 displayed stable disease, with no instances of disease progression. An impressive ORR of 81.7% was observed (**Figure 1**). Upon pathological assessment, 47 patients achieved pCR, 73 exhibited MPR, and IPR was observed in only 17 patients. Clinical to pathological downstaging was observed in 120 patients (87.6%).

Association analysis demonstrated that among patients achieving a clinical complete response, all achieved a pCR. Conversely, among patients without a clinical complete response, only 14.3% attained a pCR, signifying a significant distinction (**Table 2**, p<0.001).

**Table 2:** Efficacy of neoadjuvant immunochemotherapy in the 137 patients.

Clinical	Pathologic		
	Complete response	Major pathologic response	Incomplete pathological response
Complete response	32	0	0
Partital response	15	60	5
Stable disease	0	13	12

### Safety

A total of 621 adverse events were documented, with an average of 4.5 events per patient, but there was no long term events. Severe grade III and IV adverse events were notably rare, accounting for merely 1.6% of all reported events and were observed in 10 patients. The most prevalent adverse reactions were alopecia (100%), nausea (65.0%), and leukopenia (54.7%), whereas anemia was the least frequent adverse event (n=3, 2.2%). Severe adverse symptoms were predominantly associated with leukopenia and thrombocytopenia (**Table 3**).

**Table 3:** Grade of adverse events in neoadjuvant immunochemotherapy in the 137 patients.

Events	I/II	III/IV
Alopecia	137 (100%)	–
Nausea	89 (65.0%)	–
Leukopenia	70 (51.1%)	5 (3.6%)
Anorexia	70 (51.1%)	–
Fatigue	55 (40.1%)	–
Constipation	50 (36.5%)	–
Hypothyroidism	42 (30.7%)	–
Pain	30 (21.9%)	–
Thrombocytopenia	25 (18.2%)	5 (3.6%)
RCCEP*	14 (10.2%)	–
Fever	10 (7.3%)	–
Pneumonia	7 (5.1%)	–
Diarrhea	5 (3.6%)	–
Rash	4 (2.9%)	–
Anemia	3 (2.2%)	–

*RCCEP, reactive cutaneous capillary endothelial proliferation.

### Survival

**Figure 2:**
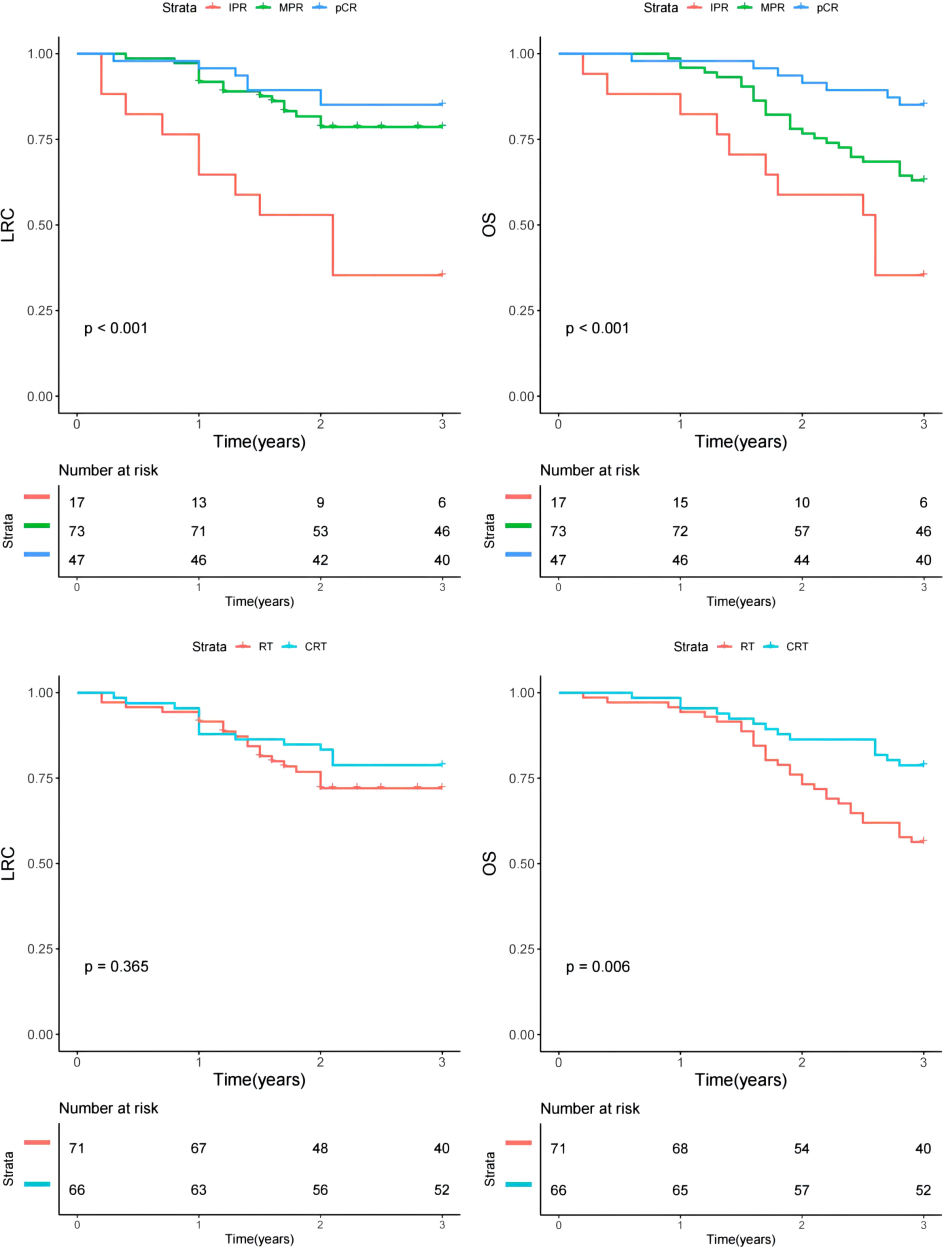
Comparison of overall survival (OS) and locoregional control (LRC) in patients with different features.

In univariate analysis, primary tumor site and treatment response significantly impacted prognosis for LRC (**Figure 2**). Subsequent multivariable analysis revealed that compared to patients with buccal or gingival tumors, those with tumors in the tongue or floor of the mouth had a hazard ratio (HR) of 2.16 (95% CI: 1.25-5.34), reflecting a significant difference (p=0.007). The HRs were 0.84 (95% CI: 0.75-0.95) for the MPR group and 0.66 (95% CI: 0.47-0.88) for the pCR classification, both significantly higher (p=0.025 and p=0.005, respectively) compared to patients with IPR. The inclusion of adjuvant chemotherapy alongside RT did not correlate with a reduced risk of locoregional recurrence (p=0.073, **Figure 2**) (**Table 4**).

**Table 4:** Univariate and multivariable analysis of predictors for locoregional control.

Variable	Univariate	Multivariable	
	p	p	HR [95%CI]
Age			
≤50			
>50	0.723		
Smoker			
Yes			
No	0.358		
Drinker			
Yes			
No	0.549		
Site			
Buccal/Gingiva			ref
Tongue/Mouth floor	<0.001	0.007	2.16 [1.25-5.34]
CPS^%^			
<1			
1-19			
≥20	0.322		
Treatment response*			
IPR			ref
MPR		0.025	0.84 [0.75-0.95]
pCR	<0.001	0.005	0.66 [0.47-0.88]
Perineural invasion			
No			
Yes	0.245		
LVI^&^			
No			
Yes	0.098		
Adjuvant therapy^			
RT			
CRT	0.073		

*IPR, Incomplete pathological response; MPR, major pathological response; pCR, pathologic complete response.

^&^LVI, Lymphovascular invasion.

^^^RT, radiotherapy; CRT, chemoradiation.

Regarding OS, primary tumor site, treatment response, and adjuvant therapy exhibited significant associations with prognosis in univariate analysis (**Figure 2**). Upon further multivariable analysis, patients with tumors in the tongue or floor of the mouth had a HR of 3.14 (95% CI: 1.53-7.36) compared to those with buccal or gingival tumors, representing a significant disparity (p=0.017). The HRs were 0.85 (95% CI: 0.73-0.96) for the MPR group and 0.66 (95% CI: 0.37-0.89) for the pCR classification, both significantly higher (p=0.018 and p<0.001, respectively) than those for patients with IPR. The addition of adjuvant chemotherapy to RT correlated with a 5% reduction in mortality risk (95% CI: 1%-14%) (**Table 5**).

**Table 5:** Univariate and multivariable analysis of predictors for overall survival.

Variable	Univariate	Multivariable	
	p	p	HR [95%CI]
Age			
≤50			
>50	0.476		
Smoker			
Yes			
No	0.813		
Drinker			
Yes			
No	0.544		
Site			
Buccal/Gingiva			ref
Tongue/Mouth floor	<0.001	0.017	3.14 [1.53-7.36]
CPS^%^			
<1			
1-19			
≥20	0.315		
Treatment response*			
IPR			ref
MPR		0.018	0.85 [0.73-0.96]
pCR	<0.001	<0.001	0.66 [0.37-0.89]
Perineural invasion			
No			
Yes	0.543		
LVI^&^			
No			
Yes	0.209		
Adjuvant therapy^			
RT			ref
CRT	0.006	0.036	0.95 [0.86-0.99]

*IPR, Incomplete pathological response; MPR, major pathological response; pCR, pathologic complete response.

^&^LVI, Lymphovascular invasion.

^^^RT, radiotherapy; CRT, chemoradiation.

### Subgroup analysis

A subgroup analysis was conducted to assess the impact of RT versus CRT on prognosis among patients stratified by pathological treatment response (**Table 6**). Patients classified with MPR displayed a 24% decreased mortality risk when treated with CRT compared to RT alone, a statistically significant finding. However, in other subgroups, CRT and RT demonstrated comparable influences on OS and LRC (all p>0.05).

**Table 6:** Subgroup analysis of the impact of adjuvant therapy on survival stratified by pathologic assessment.

Assessment*	Overall survival		Locoregional control	
	p	HR [95%CI]	p	HR [95%CI]
pCR				
RT		ref		ref
CRT	0.365	1.27 [0.83-4.29]	0.176	1.13 [0.75-6.32]
MPR				
RT		ref		ref
CRT	0.018	0.76 [0.54-0.88]	0.427	1.53 [0.82-5.43]
IPR				
RT		ref		ref
CRT	0.764	1.85 [0.36-8.90]	0.792	2.01 [0.35-9.17]

*pCR, pathologic complete response; MPR, major pathologic response; IPR, incomplete pathologic response; RT, radiotherapy; CRT, chemoradiation.

## Discussion

Our paramount discovery underscored the remarkable safety profile of neoadjuvant immunochemotherapy in the management of locally advanced oral SCC, showcasing an outstanding ORR exceeding 80% and an impressive pCR rate of 34.3%. Noteworthy, all tumors were successfully subjected to R0 resection. Notably, when juxtaposed with RT alone, the adoption of CRT yielded a superior 5% increment in OS with a 95% CI ranging from 1% to 14%, as opposed to LRC. Furthermore, both MPR and pCR emerged as robust predictors for both OS and LRC outcomes. This study serves as a pioneering effort, furnishing the initial substantiation of enhanced survival benefits conferred by neoadjuvant immunochemotherapy, thereby potentially reshaping the clinical approach to addressing locally advanced oral SCC.

The phenomenon of immune evasion serves as a pivotal driver of tumor progression, catalyzing the emergence of immunotherapy as a vanguard in the realm of oncological treatment. Notably, Nivolumab has heralded a paradigm shift in cancer therapeutics, elevating the one-year survival rate in malignant melanoma from 42.1% with conventional chemotherapy regimens to an impressive 72.9% (13). The transformative impact of Nivolumab extends across diverse malignancies, including non-small cell lung cancer, renal cell carcinoma, and head and neck SCC (14, 15). In a pivotal CheckMate-141 trial, Nivolumab showcased its prowess in treating platinum-resistant recurrent/metastatic head and neck SCC, yielding a median survival of 7.7 months—a noteworthy 2.6-month enhancement compared to the standard treatment cohort’s median survival of 5.1 months. Notably, the 2-year survival rates were substantially elevated at 16.9% for the Nivolumab group compared to 6% in the standard treatment arm, signifying a notable 32% reduction in mortality risk. These findings underscore the superiority of Nivolumab immunotherapy over conventional chemotherapy in addressing recurrent/metastatic head and neck SCC (16). Subsequent investigations such as the Keynote-040 study have corroborated these advancements, with Pembrolizumab demonstrating comparable efficacy to Nivolumab (17). This collective body of research has solidified the pivotal role of immunotherapy as a second-line therapeutic modality for managing recurrent/metastatic head and neck SCC. Moreover, landmark studies like Keynote-048 in the realm of head and neck SCC immunotherapy have shed light on the enduring benefits of Pembrolizumab monotherapy for individuals with high PD-L1 expression, showcasing noteworthy long-term survival efficacy compared to traditional treatment modalities (18). The paradigm shift towards immunotherapy, both as a first-line and second-line treatment, has been endorsed in various clinical guidelines, heralding a new era of improved outcomes and prognostic advancements in addressing recurrent/metastatic head and neck SCC. Overall, the advent of immunotherapy has revolutionized the therapeutic landscape for recurrent/metastatic head and neck SCC, presenting a compelling avenue for enhancing treatment efficacy and refining patient prognosis.

In light of the encouraging clinical outcomes witnessed with immunotherapy in recurrent/metastatic head and neck SCC, research endeavors have ventured into exploring its application in the neoadjuvant setting. Findings from a pioneering single-arm clinical study by Luginbuhl et al. (19) have illuminated the potential of neoadjuvant immunotherapy in synergy with chemotherapy. The incorporation of nivolumab alongside paclitaxel and carboplatin regimens revealed a striking pCR rate of 49%, with a combined pCR and MPR rate reaching a noteworthy 65% in locally advanced resectable head and neck SCC. Similarly, outcomes from a phase II study evaluating the neoadjuvant regimen of treprizumab combined with chemotherapy exhibited compelling advancements in pathological remission, mirroring the substantial effects observed in previous single-arm investigations combining similar regimens (20). Remarkably, the neoadjuvant therapy employing immunotherapy in conjunction with chemotherapy showcased pCR rates of 57.14% and 22.22%, respectively, with corresponding pCR+MPR rates of 92.85% and 22.22%, a tantalizing progression from prior results (21). This enhanced efficacy might be attributed to the utilization of albumin-bound paclitaxel and cisplatin regimens within the chemotherapy protocol. Studies have demonstrated the superior anti-tumor effects of protein-bound paclitaxel when combined with platinum, fluorouracil, and cetuximab in locally advanced head and neck SCC compared to conventional paclitaxel-based regimens. Notably, albumin-bound paclitaxel obviates the need for hormone pretreatment, circumventing the immunosuppressive effects of hormones and facilitating the optimal therapeutic impact of immunotherapy. This aspect may serve as a contributing factor to the enhanced rate of pathological remission observed in the neoadjuvant setting, pointing towards a promising avenue for improving treatment outcomes in this challenging clinical domain.

The side effects associated with preoperative neoadjuvant therapy incorporating immunotherapy alongside chemotherapy are predominantly manageable. Common adverse reactions encompass granulocyte deficiencies, electrolyte imbalances, nausea, and temporary hair loss. Vigilant monitoring of pertinent laboratory parameters throughout the treatment course proves instrumental in mitigating these adverse effects. Notably, in the context of this study, no instances of treatment-induced adverse reactions impeding subsequent therapeutic interventions surfaced during the neoadjuvant therapy regimen. It is noteworthy that immune-related adverse reactions, such as hyperthyroidism and hypothyroidism, manifested more frequently during the later phases of maintenance immunotherapy, underscoring the dynamic nature of immune modulation throughout the treatment continuum.

A crucial determinant in enhancing the effectiveness of immunotherapy lies in the precise identification of potential beneficiaries through robust screening methodologies. Unraveling the intricacies of biomarkers indicative of immunotherapy responsiveness stands at the forefront of research pursuits. The expression level of PD-L1 emerges as a pivotal gauge for prognosticating the efficacy of immunotherapy, with the CPS serving as a recommended predictor for immunotherapeutic outcomes in head and neck malignancies as per the NCCN guidelines (22). Notably, a CPS value equal to or exceeding 20 signifies a significant advantage in immune monotherapy efficacy, with an escalating CPS correlating with augmented prospects of responding favorably to immunotherapy. Previous investigations underscored the high incidence of PD-L1 expression in oral squamous cell carcinoma patients, with a positive rate reaching 87.88%, thus indicating the potential benefits of immunotherapy in this patient subset (20). Encouragingly, all oral cancer patients with CPS ≥ 20 achieved a MPR post neoadjuvant therapy, suggesting a substantial therapeutic benefit conferred by neoadjuvant immunotherapy in conjunction with chemotherapy. This bodes well for augmenting the long-term survival rates among this cohort of patients. Moreover, despite a subset of our patients exhibiting a CPS below 1, the overall response rate exceeding 80% underscores the imperfect predictive utility of PD-L1 alone in determining treatment efficacy. Although widely utilized immune efficacy predictors such as tumor mutation burden and microsatellite instability in head and neck SCC have not entirely met clinical exigencies in terms of accuracy, the quest continues for more precise screening biomarkers pinpointing advantageous patient populations. Exploratory endeavors into tertiary lymphoid structures within lung cancer, liver and gallbladder cancers, and malignant melanoma have unveiled their potential as autonomous predictors of immunotherapeutic outcomes (23). However, the applicability of these markers in neoadjuvant immunotherapy for locally advanced oral cancer warrants further investigation, presenting an intriguing avenue for future research pursuits.

The prognosis of locally advanced oral cancer remains a pressing concern, necessitating concerted efforts to enhance patient survival rates and overall outcomes. While induction chemotherapy may not universally bolster long-term survival in individuals with head and neck SCC, meticulous stratified analyses have unveiled a compelling narrative. Notably, following induction chemotherapy, surgical interventions yielded a commendable 10-year survival rate of 76.2% for patients achieving a pCR, starkly contrasting with the 41.3% rate observed in those falling short of the coveted pCR milestone (24). Within the realm of neoadjuvant therapy, both pCR and MPR have emerged as internationally acclaimed prognostic markers, crucial for predicting overall survival post-treatment. Pathologic remission stands as an objective and insightful yardstick for assessing the efficacy of neoadjuvant therapy, concurrently offering pivotal insights into the long-term benefits conferred (25). Leveraging the pathologic remission benchmarks elucidated in this study, it is conjectured that the innovative adjuvant protocol integrating immunotherapy with chemotherapy holds significant promise for bolstering the overall survival rates of individuals battling oral SCC. Current study data signal a promising trajectory in augmenting OS and LRC through the integration of immunotherapy with neoadjuvant chemotherapy. Post-neoadjuvant immunochemotherapy and surgical interventions remain areas warranting further exploration, with lingering uncertainties persisting regarding the optimal management strategies for patients achieving pCR or MPR milestones. At our center, surgical excision scope adherence to the initial disease stage remains standard practice, underpinned by the need for a deeper comprehension of the tumor regression patterns post-treatment. Noteworthy observations include the potentiation of overall survival through adjuvant chemotherapy complementing radiotherapy, particularly accentuated in patients achieving MPR, pioneering a novel finding in the field. Despite conventional indicators advocating chemoradiotherapy for cases featuring extranodal extension or positive margins, our pathological analyses notably omitted these factors, possibly attributable to the protracted anti-cancer effects of immunotherapy coupled with the synergistic potential of adjuvant chemotherapy.

The contrasting efficacy evaluations based on the RECIST 1.1 criteria prelude to surgery vis-à-vis postoperative pathological assessments echo a recurrent disparity observed in prior immunoneoadjuvant therapies (26). While the RECIST 1.1 standard scrutinizes tumor dimensions to gauge efficacy, the postoperative pathological appraisal delves deeper, scrutinizing residual tumor cells, necrosis levels, inflammatory responses, and tissue reactions, thus offering a comprehensive portrayal of the treatment response. It is discernible that pathological evaluation eclipses the RECIST 1.1 standard in furnishing a more nuanced understanding of tumor responsiveness, thereby furnishing a robust framework for guiding subsequent adjuvant therapeutic interventions. Future research trajectories mandate a meticulous examination of imaging attributes, extraction of features correlated with pathological assessments, refinement of image evaluation for predictive pathological responses, and an overall enhancement of clinical diagnostic and therapeutic acumen.

Limitation in current study must be acknowledged, first, there was inherent bias within the retrospective study, second, our sample size was small, it might decrease our statistic power, third, only 3-year survival was reported, longer follow-up was required.

In summary, immunochemotherapy plays an important role in the neoadjuvant treatment stage of oral cancer, achieving synergistic effects, effectively improving pathological remission and controllable safety. Pathological evaluation can objectively and accurately evaluate the effectiveness of immunotherapy, providing reliable reference for formulating adjuvant treatment plans. CRT provides better OS than RT in cases with MPR.

## Data Availability

The original contributions presented in the study are included in the article/supplementary material. Further inquiries can be directed to the corresponding author/s.
